# Molecular Identification of* Mycobacterium* Species of Public Health and Veterinary Importance from Cattle in the South State of México

**DOI:** 10.1155/2017/6094587

**Published:** 2017-06-14

**Authors:** Adrian Zaragoza Bastida, Nallely Rivero Pérez, Benjamín Valladares Carranza, Keila Isaac-Olivé, Pablo Moreno Pérez, Horacio Sandoval Trujillo, Ninfa Ramírez Durán

**Affiliations:** ^1^Facultad de Medicina, Universidad Autónoma del Estado de México, Paseo Tollocan/Jesús Carranza s/n, 50180 Toluca, MEX, Mexico; ^2^Área Académica de Medicina Veterinaria y Zootecnia, Instituto de Ciencias Agropecuaria, Universidad Autónoma del Estado de Hidalgo, Av. Universidad Km 1, Ex-Hda. de Aquetzalpa, 43600 Tulancingo, HGO, Mexico; ^3^Centro de Investigación y Estudios Avanzados en Salud Animal, Facultad de Medicina Veterinaria y Zootecnia, Universidad Autónoma del Estado de México, Km 15.5 Carretera Panamericana Toluca-Atlacomulco, 50200 Toluca, MEX, Mexico; ^4^Departamento de Sistemas Biológicos, Universidad Autónoma Metropolitana-Xochimilco, Calzada del Hueso 1100, 04960 Ciudad de México, Mexico

## Abstract

*Mycobacterium* genus causes a variety of zoonotic diseases. The best known example is the zoonotic tuberculosis due to* M. bovis*. Much less is known about “nontuberculous mycobacteria (NTM),” which are also associated with infections in humans. The Mexican standard NOM-ZOO-031-1995 regulates the presence of* M. bovis* in cattle; however, no regulation exists for the NTM species. The objective of this study was to isolate and identify nontuberculous mycobacteria species from cattle of local herds in the south region of the State of Mexico through the identification and detection of the 100 bp molecular marker in the 23S rRNA gene with subsequent sequencing of the 16S rRNA gene. Milk samples (35) and nasal exudate samples (68) were collected. From the 108 strains isolated, 39 were selected for identification. Thirteen strains isolated from nasal exudates amplified the 100 bp molecular marker and were identified as* M. neoaurum* (six strains),* M. parafortuitum* (four strains),* M. moriokaense* (two strains), and* M. confluentis* (one strain). Except* M. parafortuitum*, the other species identified are of public health and veterinary concern because they are pathogenic to humans, especially those with underlying medical conditions.

## 1. Introduction

The genus* Mycobacterium* causes a wide variety of zoonotic diseases. The best known example is zoonotic tuberculosis due to* M. bovis*, for which cattle is the main reservoir.* M. bovis* is part of the “tuberculosis complex,” which also includes the species* M. tuberculosis, M. africanum, M. caprae*, and* M. microti* [1].

Within the mycobacterial group are the “nontuberculous mycobacteria (NTM)," which are also associated with infections in humans. The NTM are found in various environmental sources such as soil, water, vegetation, animals, dairy products, and feces and may be transmitted inadvertently by inhalation, ingestion, or skin penetration [2].

The Mexican standard NOM-ZOO-031-1995 regulates the presence of* M. bovis* in cattle to control and eradicate bovine tuberculosis (bTB); however, no regulation exists for the NTM species. The official diagnosis of bovine tuberculosis due to the presence of* M. bovis* at the field level is based on the intradermal test using a purified protein derivative (tuberculin) [3]. Although used for several years, this test does not provide good sensitivity and specificity. Approximately 20% of the animals with tuberculosis do not react to the test [4], and the presence of other mycobacterial species, both tuberculosis complex and NTM species, causes interference that leads to false-positive and false-negative diagnoses.

Although Mexico has a regulatory standard, bTB prevalence in excess of 2% is reported in some areas [5]. Given the poor specificity and sensitivity of the tuberculin test, the actual presence of* M. bovis *is likely to be lower and the infection rate of cattle by other mycobacteria is likely to be higher, respectively. Thus, cattle breeders, veterinarians, technicians, and employees working in the livestock industry might be occupationally exposed to infections by* M. bovis* and NTM. Very little is known about occupational exposure to zoonoses due to NTM species because the identification of these species was a rather difficult task prior to the development of identification techniques based on molecular biology.

Currently, the molecular biology techniques most commonly used for the diagnosis of diseases caused by mycobacteria are restriction fragment length polymorphism (RFLP) for the diagnosis of* M. tuberculosis* [6], spoligotyping for the diagnosis of* M. bovis* [7], and the detection of a 100-base pair (bp) “specific insertion” located on the 23S rRNA gene characteristic of Gram-positive bacteria with a high guanine-cytosine (HGC) content, which is considered a molecular marker for this group of bacteria [8, 9], followed by sequence analysis of the 16S rRNA gene for the identification of bacteria at the species level [10].

Among the NTM species identified by the aforementioned techniques are* M. balnei, M. marinum*, and* M. platypoecilus*, which have caused superficial and deep skin lesions [11];* M. kansasii* from lung lesions [12];* M. simiae* from generalized infections [13];* M. scrofulaceum* from infections of the skin and internal organs [14];* M. szulgai* associated with pulmonary infections, osteomyelitis, tenosynovitis, and lymphadenitis [15];* M. ulcerans* associated with subcutaneous granulomas [16];* M. fortuitum* and* M. chelonae* associated with vasculitis, endocarditis, osteomyelitis, mediastinitis, meningitis, keratitis, and hepatitis [17];* M. abscessus*, associated with erythematous lesions that progressed to ulcerated nodules [18]; and other species.

The largest percentage of the state inventory for heads of cattle in the State of Mexico in Mexico is concentrated in the southern region, and one of the main economic activities is cattle ranching [19]. The Mexican regulation for cattle control NOM-ZOO-031-1995 only focuses on the tuberculin test for the diagnosis of* M. bovis*. Little is known about the presence of NTM in the cattle of the region. Given the possibility of identifying species of actinobacteria by detection of the 100-base pair molecular marker on the 23S rRNA gene and the subsequent sequencing of the 16S rRNA gene, it is possible to identify the aforementioned NTM species.

The objective of the present study was to isolate and identify NTM species from cattle of the south region of the State of Mexico. The* Mycobacterium* species were isolated from samples of nasal exudate and bovine milk and identified by detecting the 100-base pair molecular marker in the 23S rRNA gene with subsequent sequencing of the 16S rRNA gene.

## 2. Materials and Methods

### 2.1. Sampling

A sampling was performed based on the spatial distribution of herds positive for bovine tuberculosis in the state of Mexico conducted by Zaragoza et al. 2015 [20]. Four herds of cattle were selected in the south region of the State of Mexico, one herd belonging to the Municipality of Temascaltepec and three herds belonging to the municipality of Zacazonapan. A total of 103 samples, 35 milk samples and 68 samples of nasal exudate, were collected. The distribution of the number and type of samples collected in each herd is shown in Table 1.

### 2.2. Obtaining Samples of Milk and Nasal Exudate

The udder and nipples were cleansed with purified water and soap and then dried with paper towels, and nipple asepsis was subsequently performed using swabs soaked in 70% alcohol. Five milliliters of milk was collected directly from the nipple in sterile 20 mL vessels, discarding the initial flow. Nasal exudate was collected directly from the inside of the nasal orifice using a 10 cm long sterile swab, which was then submerged in an isotonic saline solution (0.85%). Samples of milk and nasal exudate were stored at 4°C until processing.

### 2.3. Sample Processing

#### 2.3.1. Isolation of Mycobacteria

The milk samples were centrifuged at 2500 revolutions per minute (rpm) for 10 minutes. The pellets from the milk and nasal exudate samples were inoculated into the following culture medium selective for mycobacteria: Stonebrink (BD BBL 220504), Middlebrook (BD BBL 254521), and Middlebrook (BD BBL 254521) supplemented with 6 g of sodium pyruvate per liter (Middlebrook-P). The inoculated media were incubated at 37°C for 8 weeks and were assessed every 3 days.

#### 2.3.2. Classification of Isolated Strains

The isolated strains were distributed in groups according to the following characteristics: colony pigmentation, growth time, and colony characteristics (shape, consistency, texture, and pigment production). Isolated strains were stained with Ziehl-Neelsen to confirm the presence of acid-fast bacilli (AFB) [21].

### 2.4. DNA Extraction

Strains with microscopic characteristics similar to mycobacteria (acid-fast positivity) and two representative strains of each group were selected for identification. To obtain biomass, the strains were inoculated into 30 mL of Middlebrook liquid culture medium (BD BBL 254521) in 125 mL flasks and incubated at 37°C for 7 days. The liquid medium was transferred to sterile 15 mL Falcon tubes and centrifuged for 15 minutes at 14,000 rpm. Then, the supernatant was removed and the pellet was transferred to 1.5 mL Eppendorf tubes; the tubes were then centrifuged at 14,000 rpm × 5 minutes, and the supernatant was discarded. DNA extraction was performed on the resulting pellet using the Wizard Genomic DNA Purification kit (Promega A1120).

### 2.5. Detection of the Molecular Marker in the 23S rRNA Gene

The 100 bp molecular marker located on the 23S rRNA gene was amplified according to the methodology described by Roller et al. (1992) using the following primers [8]:

  23S InsF, 5′-(AC)A(AGT)GCGTAG(AGCT)CGA(AT)GG-3′, and 23S InsR, 5′-GTG(AT)CGGTTT(AGCT)(GCT)GGTA-3′.

The reaction was conducted using a commercial Taq DNA polymerase (Promega M1661). The following thermal cycle conditions were used: a predenaturation step for 5 minutes (94°C); 29 cycles of denaturation for 30 seconds (94°C), hybridization for 45 seconds (46°C), and elongation for 50 seconds (72°C); and, finally, a postelongation cycle of 5 minutes (72°C). The amplified fragments were confirmed on a 2% agarose gel stained with ethidium bromide (SIGMA 46065).

### 2.6. Amplification of the 16S rRNA Gene

Strains that amplified the 100 bp phylogenetic marker were selected for 16S rRNA sequencing analysis. The following primers were used for the amplification:

  8f: AGAGTTTGATCMTGGCTCAG and 1492r: TACGGYTACCTTGTTACGACTT.

The reaction was conducted using a commercial Taq DNA polymerase (Promega M1661). The following thermal cycle conditions were used: one predenaturation step for 5 minutes (94°C); 34 cycles of denaturation for 30 seconds (94°C), hybridization for 20 seconds (52°C), and elongation for 1 minute 30 seconds (72°C); and, finally, a postelongation cycle of 7 minutes (72°C).

The amplified fragments were confirmed on a 1% agarose gel stained with ethidium bromide (SIGMA 46065). The products of this amplification were purified using the Amicon Ultra Filter® kit (Millipore UFC901008) and confirmed on a 1% agarose gel to verify their presence and quality.

### 2.7. Identification of* Mycobacterium* Species

The amplified products of the 16S rRNA gene were sent to the Macrogen Sequencing Service, Maryland, USA. The obtained sequences were analyzed and corrected using the BioEdit program [22]. Consensus sequences were constructed from the forward and reverse fragments, which were compared with sequences deposited previously in GenBank of the National Center for Biotechnology Information (NCBI) using the BLAST program [23] and EzTaxon 2.1 [24].

### 2.8. Phylogenetic Analysis

Sequences of the 16S rRNA gene were obtained for the following mycobacterial species from the American Type Culture Collection (ATCC) and the German Collection of Microorganisms and Cell Cultures (DSM):* M. neoaurum* ATCC^25795^,* M. parafortuitum* DSM^43528^,* M. moriokaense *DSM^44221T^, and* M. confluentis *DSM^44017T^. The sequences of the collection strains and those of the strains isolated in the present investigation were aligned with the BioEdit program [22]. Phylogenetic analysis was performed using the maximum parsimony method in MEGA software version 4 [25]. To form the root of the cladogram, the sequence of* Pantoea agglomerans* DSM 3493 was used.

## 3. Results

The 108 strains isolated from the 103 collected samples were distributed in 13 groups according to their macroscopic and microscopic morphological characteristics (Table 2). Groups 11 and 12, particularly, were composed of acid-fast strains.

For identification at the species level, 39 strains were chosen: 10 of them belonged to group 11 and 7 to group 12. Two strains from each one of the remaining 11 groups were selected to complete the 39 strains. The 100 bp molecular marker was found in the 33% (13/39) of the selected strains. For them, the 16S rRNA gene was amplified for sequencing and identification at the species level.

The overall prevalence of NTM on the collected samples was 12.6% (13/103) considering both milk and nasal exudate samples. However, the specific prevalence for nasal exudate samples was 19.1% (13/68).

According to the sequence comparison, four NTM species of the genus* Mycobacterium* were identified; 64% (6/13) of the strains had 98% and 99% of similarities with* M. neoaurum*, while 31% (4/13) had 99% similarity with* M. parafortuitum*, 15% (2/13) had similarities of 98% and 99% with* M. moriokaense*, and, finally, 8% (1/13) had 99% similarity with* M. confluentis* (Table 3).

**Figure 1:**
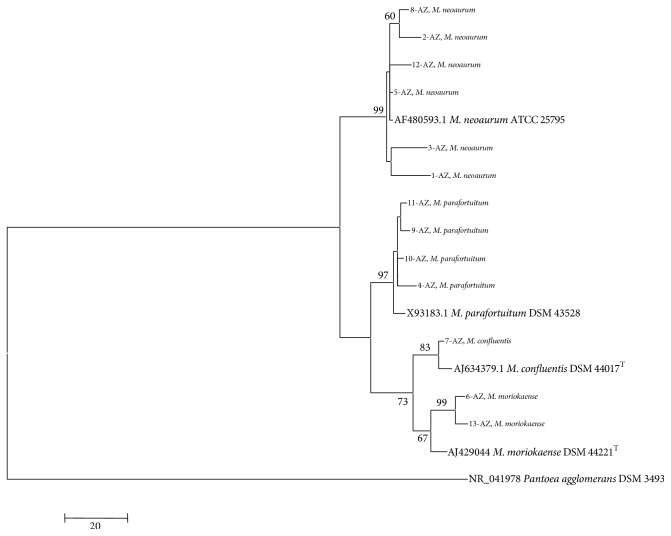
Phylogenetic tree constructed by comparing the 16S rRNA gene sequences from the isolated and reference strains.

The phylogenetic tree was formed with the genus* Mycobacterium* and four of its species by which the phylogenetic relationships between the collection strains and the strains isolated in the present investigation were observed (Figure 1).

## 4. Discussion

The NTM species were isolated from samples of nasal exudate only, which eliminated the samples from one of the local farms of this study (Table 1). We found that the specific prevalence was 19.1% in herds of the south region of the State of Mexico. Similar studies in the United States, South Africa, Tanzania, and Brazil reported NTM prevalence values of 3.4%, 24.5%, 7%, and 7.8%, respectively; therefore, the prevalence value found in this study lies within the range reported previously [26–29]. In this study, 13 of the 39 analyzed strains were identified as the NTM species* M. neoaurum, M. moriokaense, M. confluentis*, and* M. parafortuitum*.

*M. neoaurum*, a member of the* Mycobacterium parafortuitum* complex, is responsible for a broad spectrum of illnesses, most of them device related infections such as Hickman catheters, BROVIAC catheters, PICC lines [30–33], arteriovenous fistula that included a polytetrafluoroethylene graft [34], pace makers [35], and prosthetic valve endocarditis [36]. Immunocompromised patients holding these devices are the principal hosts, for example, patients suffering from cancer [32] and diabetics with renal failure [31, 33, 34] and heart problems [35].* M. neoaurum* has also been isolated from patients with urinary infections [37], meningoencephalitis and alterations in the central nervous system [38], bacteremia and endocarditis [39], and pulmonary infection [40, 41]. Although it has been mainly isolated from clinical cases, there are also reports about its isolation from milk and cattle [28, 42, 43].

*M. moriokaense* was isolated from sputum sample [44]. Although it is considered nonpathogenic for humans, it has been associated with pulmonary diseases [45].* M. confluentis* was isolated from sputum samples as well [46], and, along with* M. parafortuitum*, both are considered nonpathogenic species.* M. confluentis, M. moriokaense*, and* M. neoaurum* have been isolated from different bovine and wildlife tissues with tuberculous lesions, whereas* M. parafortuitum* has only been isolated from bovine milk [26, 28, 47–49]. However, in our work,* M. parafortuitum* was only isolated from nasal exudate samples.

The nutritional requirements of mycobacteria differ among various species, which was the reason for using different culture media. Notably, seven of the 13 strains identified in this study were isolated in Stonebrink medium, including* M. neoaurum, M. parafortuitum, *and* M. moriokaense*. This result is consistent with that described by Sepúlveda et al. [50] who indicated that Stonebrink medium is suitable for the recovery of different species of the genus* Mycobacterium*. García-Martos and García-Agudo [51] reported that Middlebrook medium is optimal for the isolation of actinomycetes, which is in accord with the present investigation considering that two species,* M. neoaurum* and* M. parafortuitum*, were isolated in this medium. Notably,* M. confluentis* was isolated only in Middlebrook medium supplemented with sodium pyruvate; thus, the strategy of using different culture media was appropriate because it allowed the isolation of different species of the genus* Mycobacterium*.

The detection of the molecular marker present in the 23S rRNA gene of Gram-positive bacteria with HGC content allowed discrimination between strains of eubacteria and mycobacteria. The sequencing analysis of the 16S rRNA gene made the identification at the species level possible; therefore, the combination of these methodologies is appropriate for the identification of NTM species.

## 5. Conclusions

Using the methodology described in this study, four NTM species were isolated and identified:* M. confluentis, M. moriokaense, M. neoaurum,* and* M. parafortuitum*. These species were isolated for the first time from nasal exudates of bovines from the south region of the State of Mexico. Three of the identified species (*M. neoaurum, M. moriokaense,* and* M. confluentis*) are of public health and veterinary importance.

## Figures and Tables

**Table 1:** Samples collected in cattle herds in the south region of the State of Mexico.

Characteristic	Herd 1	Herd 2	Herd 3	Herd 4	Total
Municipality	Temascaltepec	Zacazonapan	Zacazonapan	Zacazonapan	
Breed	F1 Swiss-Cebu	Holstein Friesian	Holstein Friesian	Holstein Friesian	
Geographic location	La-19°03′13.7′′Lo-100°13′36.7′′	La-19°03′39.5′′Lo-100°16′30.9′′	La-19°04′0.4′′Lo-100°15′11.5′′	La-19°03′41′′Lo-100°16′06′′	
History of bTB	Prevalence 0.2%^*∗*^	Prevalence 0.2%^*∗*^	Prevalence 0.2%^*∗*^	Prevalence 0.2%^*∗*^	

Samples obtained					

Milk	15	20	0	0	35
Nasal exudate	0	18	23	27	68
					**103**

La: latitude; Lo: longitude; bTB: bovine tuberculosis. ^*∗*^Information obtained from the Committee on the Promotion and Protection of Livestock of the State of Mexico.

**Table 2:** Isolated strains are grouped according to their morphological characteristics and the presence of the molecular marker (100 bp) on the 23S rRNA gene.

Group	Number of Strains	Morphological characteristics			Molecular marker (bp)
		Macroscopic		Microscopic	23S rRNA
		Pigmentation	Appearance	Ziehl-Neelsen	
1	29	Yellow	Creamy	−	250
2	10	White	Creamy	−	250
3	14	White	Dry	−	250
4	2	Salmon	Creamy	−	250
5	2	Salmon	Dry	−	250
6	2	White, dark	Creamy	−	250
7	2	White	Creamy	−	250
8	3	White, dark	Dry	−	250
9	4	White	Dry	−	250
10	10	White	Creamy, dry	−	250
11	10	White	Dry	+	350 and 250
12	7	Yellow	Creamy	+	350
13	13	White	Dry	−	250

−: absence of acid-fast bacilli; +: presence of acid-fast bacilli; Bp: base pairs.

**Table 3:** Comparison of 16S rRNA gene sequences of strains isolated from cattle with those documented in GenBank, using BLAST and EzTaxon.

Strain	Origin of the herd	Culture medium	Amplified fragment size (bp)	Similarity (Blast)	%	Similarity (EzTaxon)	%
1-AZ	2	Middlebrook	1428	*M. neoaurum*	98	*M. neoaurum*	98.3
2-AZ	2	Stonebrink	1408	*M. neoaurum*	99	*M. neoaurum*	99.1
3-AZ	2	Stonebrink	1428	*M. neoaurum*	98	*M. neoaurum*	98.2
5-AZ	3	Stonebrink	1416	*M. neoaurum*	99	*M. neoaurum*	99.2
8-AZ	4	Middlebrook	1415	*M. neoaurum*	99	*M. neoaurum*	99.0
12-AZ	2	Middlebrook	1415	*M. neoaurum*	99	*M. neoaurum*	99.4
4-AZ	4	Middlebrook-P	1420	*M. parafortuitum*	99	*M. parafortuitum*	98.2
9-AZ	3	Middlebrook	1415	*M. parafortuitum*	99	*M. parafortuitum*	98.9
10-AZ	4	Stonebrink	1411	*M. parafortuitum*	99	*M. parafortuitum*	98.4
11-AZ	3	Stonebrink	1414	*M. parafortuitum*	99	*M. parafortuitum*	98.2
6-AZ	2	Stonebrink	1455	*M. moriokaense*	99	*M. moriokaense*	98.2
13-AZ	4	Stonebrink	1417	*M. moriokaense*	98	*M. moriokaense*	98.2
7-AZ	2	Middlebrook-P	1420	*M. confluentis*	99	*M. confluentis*	99.1

2: Zacazonapan Holstein-F; 3: Zacazonapan Holstein-F; 4: Zacazonapan Holstein-F.
